# Bronchial hyperresponsiveness and asthma during oral immunotherapy for egg or peanut allergy in children

**DOI:** 10.1002/clt2.12203

**Published:** 2022-10-10

**Authors:** Janne Burman, Kati Palosuo, Anna Pelkonen, Pekka Malmberg, Sami Remes, Kaarina Kukkonen, Mika J. Mäkelä

**Affiliations:** ^1^ Skin and Allergy Hospital Helsinki University Hospital and University of Helsinki Helsinki Finland; ^2^ Department of Pediatrics Kuopio University Hospital Kuopio Finland

**Keywords:** asthma, bronchial hyperresponsiveness, food allergy, methacholine challenge test, oral immunotherapy

## Abstract

**Background:**

Bronchial hyperresponsiveness (BHR) and asthma are frequently present in children with food allergy. We assessed BHR in children receiving oral immunotherapy (OIT) for persistent egg or peanut allergy and examined whether OIT affects asthma control.

**Methods:**

Methacholine challenge testing was performed in 89 children with persistent egg or peanut allergy diagnosed by double‐blind, placebo‐controlled food challenge and 80 control children without food allergy. Of the 89 food‐allergic children, 50 started OIT for egg allergy and 39 for peanut allergy. Sensitization to aeroallergens was evaluated by skin prick testing. Forty of the 89 children with regular controller treatment for asthma underwent methacholine challenge testing and 34 measurement of exhaled nitric oxide (FeNO) at baseline and after 6–12 months of OIT.

**Results:**

Methacholine challenge testing revealed significant BHR in 29/50 children (58%) with egg allergy, 15/39 children (38%) with peanut allergy, and 6/80 controls (7.5%). The mean cumulative dose of methacholine causing a 20% fall in FEV1 differed significantly between the egg and peanut‐allergic versus the control children (1009 μg, 1104 μg, and 2068 μg, respectively, *p* < 0.001). Egg or peanut OIT did not affect lung function, the degree of BHR or FeNO levels in children with asthma and had no adverse effect on asthma control. Lung function or BHR did not associate with the OIT outcome.

**Conclusion:**

BHR was significantly more frequent in children with persistent egg or peanut allergy than in children without food allergy. Oral immunotherapy did not increase BHR and was safe for children on regular asthma medication.

## 1 INTRODUCTION

Food allergies affect approximately 8%–10% of children in the Western countries.^1^, ^2^ The worldwide reported prevalence of egg allergy in children is 1.3%–10%^1^, ^3^ and of peanut allergy 1.4%–5%.^1^, ^3^ Egg allergy resolves by school‐age in most children, while peanut allergy tends to persist.^3^ Milk and egg allergy are the most frequent causes of anaphylaxis in children, while peanut allergy is a common cause of anaphylactic reactions in all age groups.^4^, ^5^

The frequency of asthma in the European Union countries is approximately 8%–9%.^6^ Sensitization to foods increases the risk of asthma^7^, ^8^ and bronchial hyperresponsiveness (BHR)^9^, ^10^, ^11^ as well as the level of exhaled nitric oxide (FeNO).^7^, ^9^ Asthma symptoms are more prevalent in subjects with severe food allergy, and on the other hand, sensitization to foods is higher in children with asthma than in the general population.^12^, ^13^, ^14^, ^15^ Up to 50% of patients with severe food allergy have asthma and the prevalence of uncontrolled asthma is higher in these patients.^14^ Severe asthma is a risk factor for food‐related anaphylaxis because it associates with serious reactions causing bronchoconstriction in food‐allergic patients^5^, ^16^, ^17^, ^18^ Asthma is associated with 56%–78% of cases of fatal anaphylaxis to peanuts.^18^ Children with a history of an anaphylactic reaction to foods have asthma more frequently than in children with milder allergic reactions.^19^ Adequate asthma control is essential for children with food allergy as the coexistence of asthma and food allergy may negatively influence the severity of both conditions.

Oral immunotherapy (OIT) is an experimental treatment for persistent food allergy which can desensitize 75%–85% of children with egg or peanut allergy.^20^ A subset of patients achieve or long‐term immune tolerance or sustained unresponsiveness which is defined as antigen hyporesponsiveness regardless of regular intake.^2^, ^20^ Uncontrolled or severe asthma is a contraindication for OIT, and suboptimal asthma control may increase allergic reactions during OIT.^2^

Bronchial hyperresponsiveness can be assessed by direct bronchoprovocation tests (methacholine and histamine challenge), which act directly on specific airway smooth muscle receptors, or indirect stimuli (physical exercise, hyperventilation), which release mediators that provoke smooth muscle cells.^21^ The provocative dose of methacholine causing a 20% decrease in FEV1 is age‐dependent which complicates the use of methacholine challenge tests in children under 12 years^22^

Studies examining the effect of OIT on BHR and asthma control in children are limited. We have previously reported that peanut OIT in 39 children had no negative effects on lung function, FeNO, or BHR.^23^ This study aimed to examine the rate of BHR in children with persistent egg or peanut allergy compared to non‐allergic controls and to investigate differences in lung function, BHR, and FeNO and in children with asthma receiving OIT for egg or peanut allergy.

## 2 METHODS

The primary outcome was assessment of BHR by methacholine challenge testing in egg and peanut‐allergic children compared to children without food allergy. Secondary outcomes included investigation of differences in BHR, lung function, and FeNO between desensitized and failed patients receiving egg or peanut OIT, as well as the effect of OIT on BHR and emergency department visits in children with asthma.

### 2.1 Study population

**FIGURE 1:**
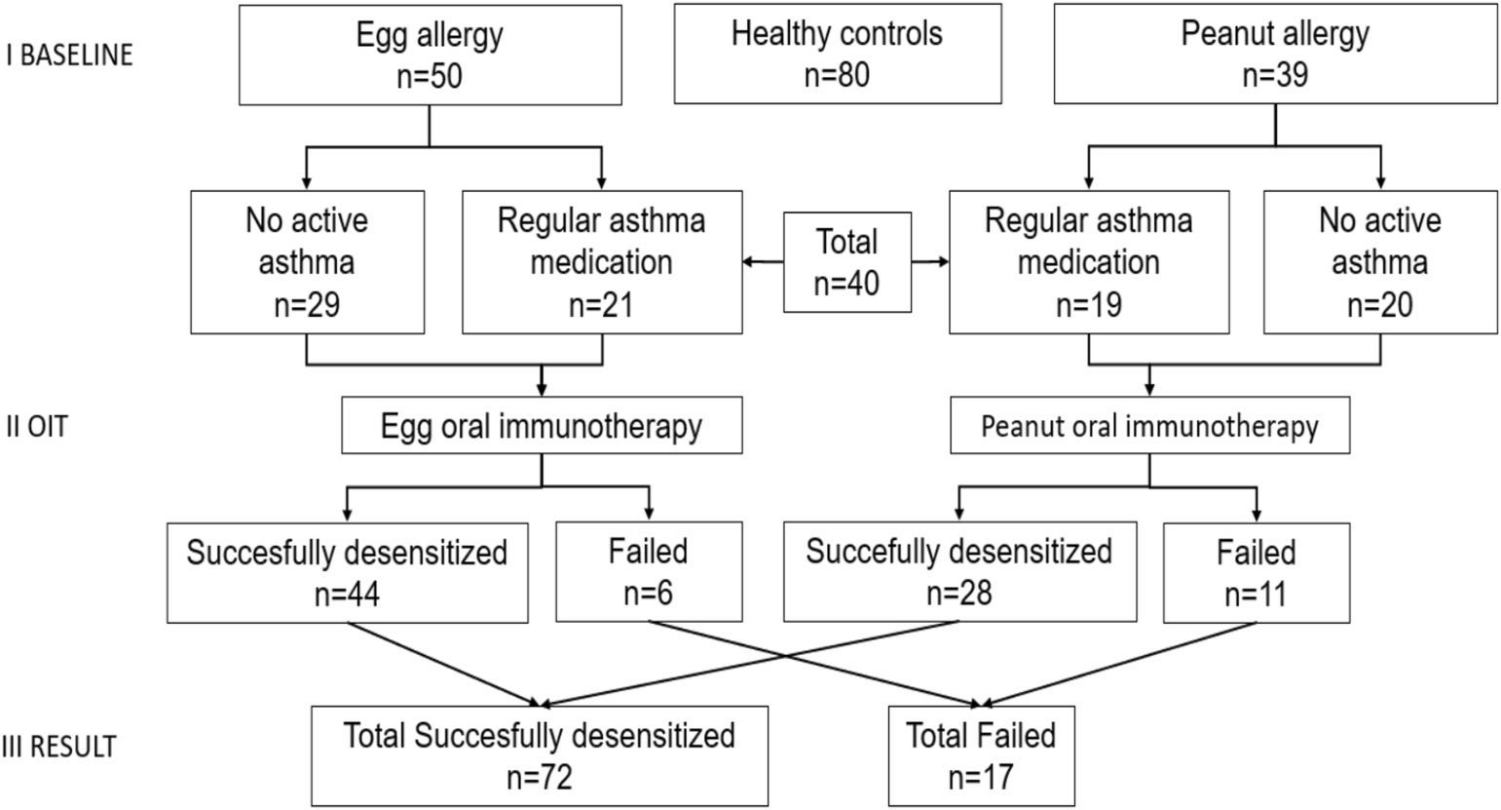
Flowchart of the study

The study included 89 children aged 6–17 years from the Helsinki University Skin and Allergy Hospital, Finland, with moderate to severe reactions^23^ to egg (*n* = 50) or peanut (*n* = 39) in a double‐blind, placebo‐controlled food challenge (DBPCFC) (Figure 1). The median cumulative protein dose was 455 mg (5–1777 mg) in children with egg allergy and 55 mg (5–1255 mg) in children with peanut allergy. Forty of the 89 children (45%) had asthma. Asthma diagnosis was based on the presence of respiratory symptoms consistent with asthma combined with the presence of variable expiratory airflow obstruction in lung function tests according to the Finnish National Guidelines.^24^ The 39 peanut‐allergic children (56% boys, mean age 9.4 years) were recruited between 2011 and 2013 and the 50 egg‐allergic children (46% boys, mean age 11.2 years) between 2013 and 2017. The Helsinki University Hospital of Children and Adolescents Ethics Committee approved the study and each participant above 6 years of age as well as his/her guardian gave written informed consent. The inclusion criteria for the study were: age 6–17 years, a clinical history of egg or peanut allergy, sensitization to egg white or peanut (specific IgE ≥0.35 kU/l), and a moderate to severe reaction in the DBPCFC. The exclusion criteria were: poor adherence, uncontrolled or severe asthma, severe systemic illness, active autoimmune disease, malignant neoplasia, or pregnancy. Eighty healthy children without food allergy aged 7–12 years (55% boys, mean age 10.4 years) from the University Hospital of Kuopio, Finland served as controls.

The families completed a questionnaire on the child's previous medical history, including asthma diagnosis in childhood ever, other allergies, and current medication. Details on previous allergic reactions to foods and the use of rescue medication were collected from the medical records.

### 2.2 Oral immunotherapy

Oral immunotherapy for egg or peanut allergy was carried out as previously described^23^, ^25^ with pasteurized, spray‐dried, raw egg white powder (Dava Foods, Piispanristi, Finland) or with roasted defatted peanut flour (Byrd Mill, Ashland, VA, USA) and whole peanuts from week 20 with daily dosing at home. The build‐up phase lasted for 8 months (32 weeks for egg and 34 weeks for peanut). The target maintenance doses were 1 g of egg white protein, corresponding approximately to one‐third of an egg‐white or four whole peanuts containing approximately 800 mg protein. We defined successful desensitization as the ability to consume the culprit food regularly (desensitized to the target dose or partially desensitized to a lower dose) without symptoms after 18 months of OIT. The patients who discontinued OIT were defined as failed. Both studies were registered at Clinicaltrials.gov (NCT01502878 and NCT03744325).

### 2.3 Allergen‐specific IgE and skin prick tests

We measured blood eosinophil levels and specific IgE to egg white and Gal d 1–4 in patients with egg allergy, and to peanut, and Ara h 2 in patients with peanut allergy by ImmunoCAP (Thermo Fisher, Uppsala, Sweden). We performed skin prick tests (SPT) with peanut, raw egg, birch, timothy, cat, dog, and house dust‐mite (ALK, Horsholm, Denmark) as previously described.^23^

### 2.4 Methacholine challenge and FeNO

We performed baseline spirometry tests according to the European Respiratory Society criteria.^26^ All 89 food‐allergic children performed a methacholine challenge test with a cumulative dose of 2600 μg methacholine as previously described.^27^ The controls performed a methacholine challenge test at the University Hospital of Kuopio, Finland^28^ where the cumulative dose of methacholine was 4800ug. Bronchoconstriction occurring at a dose above 2600ug of methacholine was registered as 2600ug. Bronchial hyperresponsiveness was considered mild if the cumulative methacholine dose causing at least a 20% fall in FEV1 was more than 600 μg and significant if it was 600 μg or less.^29^

The concentration of fractional exhaled nitric oxide (FeNO) was examined in the 89 food‐allergic children following the international guidelines as previously described.^30^ 34 of the 40 children with asthma who underwent OIT repeated methacholine challenge tests and FeNO measurements after 6–12 months of OIT.

### 2.5 Severe asthma exacerbations

To assess asthma control and the number of severe exacerbations during OIT in the 40 children with asthma, we reviewed the medical records 12 months before and after the beginning of OIT. We examined the records of the asthma follow‐up visits at the outpatients' department as well as any acute asthma‐related emergency room visits.

### 2.6 Statistical analysis

SPSS version 22 (IBM Corp, Armonk, NY, USA) was used for the statistical analysis. For continuous parametric and nonparametric data, the Student's *t*‐test and Mann‐Whitney *U* test or the Kruskal Wallis test were used, respectively. For categorical data, we used the chi‐square test, Fisher's exact test for counts of less than five, and McNemar's test for related samples. Statistical significance was *p* < 0.05.

## 3 RESULTS

### 3.1 Subject characteristics

The study enrollment is shown in Figure 1 and the baseline characteristics of the study subjects in Table 1. The children with peanut allergy were younger than the children with egg allergy or the controls (9.4 vs. 11.2 vs. 10.4 years, *p* = 0.003). There were no significant differences between the children with egg and peanut allergies in gender or the percentage of atopic eczema, asthma, or allergic rhinitis (Table 1). Altogether 40 of the 89 children (45%) who started OIT for egg or peanut allergy had doctor‐diagnosed asthma with regular controller medication (Figure 1). None of the controls had asthma and they had less frequently allergic rhinitis and atopic eczema (Table 1).

**TABLE 1:** Subject characteristics

Value	Children with egg allergy (*n* = 50)	Children with peanut allergy (*n* = 39)	*p*‐value (between egg and peanut allergy)	Controls (*n* = 80)	*p*‐value (between all groups)
Average age (years)	11.2 (3.3)	9.4 (3.5)	**0.013**	10.4 (1.6)	**0.003**
Boys	23 (46)	22 (56)	0.330	44 (55)	0.526
Atopic eczema^a^	37 (74)	26 (67)	0.450	19 (24)	**0.001**
Asthma^a^	21 (42)	19 (50)	0.455	0 (0)	**<0.001**
Allergic rhinitis^a^	39 (78)	26 (67)	0.232	18 (23)	**<0.001**

*Notes*: Data show the means (standard deviations) or the numbers of children (percentages). Calculated using chi‐square test, independent samples t‐test, and Kruskal‐Wallis test, as appropriate. Bold *p*‐values indicate *p* < 0.05.

^a^ Doctor diagnosed.

### 3.2 Specific IgE and skin prick tests

The median egg white specific IgE was 26.6 (1.0–1200.0) kU/L in the 50 children with egg allergy and the median peanut specific IgE was 74.6kU/l (1.8–1818.2) kU/L in the 39 children with peanut allergy.^23^, ^25^ The egg white or peanut specific IgE levels did not differ significantly between asthmatic and non‐asthmatic children, nor did we observe any significant correlation between egg or peanut specific IgE levels and BHR.

The egg‐ and peanut‐allergic children had similar aeroallergen sensitization profiles in SPT. Aeroallergen sensitization or the presence of allergic rhinitis did not correlate with BHR. Sensitization to at least two aeroallergens was present in 84/89 (94%). Sensitization to birch, timothy, dog, and cat was common, while house dust mite sensitization was rare. The children with egg allergy were more frequently sensitized to dog compared to children with peanut allergy (91% vs. 77%, *p* = 0.028). Of the children with egg allergy, 36/50 (72%) were sensitized to peanut, whereas 11/39 (28%) of the children with peanut allergy were sensitized to egg (Table 2). Clinical egg allergy was present in only two of the 11 children with peanut allergy.

**TABLE 2:** Laboratory findings in children with egg or peanut allergy

Value	Children with egg allergy (*n* = 50)	Children with peanut allergy (*n* = 39)	*p*‐value
Eos, E109/l	0.49 (0.29)	0.59 (0.29)	0.088
Sensitization to aeroallergen	49 (98)	38 (97)	0.650
Multisensitization to aeroallergens	48 (96)	36 (92)	0.650
Sensitization to at least 4 aeroallergens	30 (60)	19 (49)	0.288
Sensitization to all 5 aeroallergens	3 (6)	2 (5)	0.617
Sensitization^a^ to: Birch	43 (86)	34 (87)	0.872
Timothy	34 (68)	27 (69)	0.901
Dog	47 (91)	30 (77)	**0.028**
Cat	43 (86)	27 (69)	0.055
House dust mite	4 (8)	4 (10)	0.726
Raw egg	50 (100)	11 (28)	**<0.001**
Peanut	36 (72)	39 (100)	**<0.001**

*Notes*: The data represents the numbers (percentages) of children. Calculated using chi‐square test, Fisher exact test and independent samples *t*‐test, as appropriate. Bold *p*‐values indicate *p* < 0.05.

Abbreviation: Eos, blood eosinophils

^a^ sensitization defined as a reaction of a minimum of 3 mm reaction in the skin prick test.

### 3.3 Oral immunotherapy outcome

After 18 months of OIT, a total of 72/89 (81%) children were successfully desensitized, while 17 discontinued (Figure 1). Desensitization was achieved in 44/50 (88%) of the children receiving egg OIT and 28/39 (72%) of the children receiving peanut OIT. Age, gender, or the proportion of children with asthma or allergic sensitization did not differ between the groups (Table 3).

**TABLE 3:** Comparison of the study children by the egg or peanut oral immunotherapy (OIT) outcome after 18 months

Value	Successfully desensitized (*n* = 72)	Failed (*n* = 17)	*p*‐value
Age (years)	10.3 (3.4)	11.0 (3.9)	0.471
Boys	34 (47)	11 (65)	0.195
Atopic eczema^a^	52 (72)	11 (65)	0.540
Asthma^a^	32 (44)	8 (47)	0.882
Other food allergies	46 (64)	12 (71)	0.602
FEV1, (% of reference)	95.3 (10.8)	92.9 (11.3)	0.411
FVC, (% of reference)	98.2 (10.6)	95.6 (9.0)	0.369
PD‐20FEV1, ug	552 (18–2600)	706 (36–2600)	0.704
Significant hyperresponsivenesss	37 (51)	7 (41)	0.449
FeNO, ppb	17.8 (4.6–130.8)	21.9 (1.5–75.1)	0.595
FeNO minimum 35ppb	20 (28)	6 (35)	0.540
Eos, 10E9/L	0.53 (0.32)	0.53 (0.20)	0.975
Sensitization to aeroallergens	71 (99)	16 (94)	0.347
Sensitization to birch	64 (89)	13 (76)	0.178
Sensitization to house dust mite	6 (8)	2 (12)	0.656

Abbreviations: FEV1, Forced expiratory volume in 1 s; FVC, Forced vital capacity; PD‐20FEV1, the cumulative dose of methacholine (ug) causing a 20% fall in FEV; FeNO, Exhaled lower respiratory nitric oxide; Eos, blood eosinophils; Significant hyperresponsiveness defined as PD‐20FEV1 of the maximum 600 μg of methacholine, The data represents the means (standard deviations); median and range, or the numbers (percentages) of children.

^a^ Doctor diagnosed.

### 3.4 Lung function and bronchial hyperresponsiveness

The baseline lung function was similar in the children with egg or peanut allergy and the controls (Table 4). None of the children experienced significant improvement in the bronchodilatation test. Methacholine challenge testing demonstrated significant BHR in 29/50 children (58%) with egg allergy, 15/39 children (38%) with peanut allergy, and 6/80 controls (7.5%). The median cumulative dose of methacholine that caused a 20% fall in FEV1 was 1009, 1104, and 2068 μg, respectively (*p* < 0.001). Of the 44 food‐allergic children with BHR, 19 (43%) had asthma, while 25 (57%) had asymptomatic BHR. Of the 80 controls, asymptomatic BHR was observed in 6/80 (7.5%). BHR was significantly more frequent, and the median cumulative methacholine dose was markedly lower in children with egg and peanut allergy compared to the healthy controls (*p* < 0.001), but the differences between the egg‐ and peanut‐allergic children were statistically insignificant (*p* = 0.067 and 0.675). In a subanalysis of the 49 food‐allergic children without asthma, BHR was significantly more frequent (51.0% vs. 7.5%, *p* < 0.001) and the median cumulative methacholine dose significantly lower (655 μg vs. 2601 μg, *p* < 0.001) compared to the controls. Likewise, the FeNo levels (29.3 vs. 26.7ppb, *p* = 0.642) and the proportion of children having a FeNO above 35ppb (32% vs. 26%, *p* = 0.553) showed no significant differences between the children with egg and peanut allergy (Table 3). There were no differences in lung function, the presence of BHR, PD20FEV1 or FeNo levels between the 72 successfully desensitized children and the 17 children who discontinued OIT (Table 4). Bronchial hyperresponsiveness was more frequent in children with allergic rhinitis and food allergy compared to children with allergic rhinitis only (PD20FEV1 670 ug vs. 2100 ug, *p* = 0.030).

**TABLE 4:** Lung function results in children with egg and peanut allergy

Value	Children with egg allergy (*n* = 50)	Children with peanut allergy (*n* = 39)	*p*‐value between egg and peanut groups	Controls	*p*‐value between all groups
FVC, (% of reference)	97.8 (11.0)	97.6 (9.5)	0.917	96.5 (10.5)	0.812
FEV1, (% of reference)	94.4 (11.2)	95.5 (10.5)	0.621	95.7 (10.3)	0.775
Obstruction in spirometry^a^	7 (16%)	4 (10%)	0.448	5 (6.3%)	0.203
PD‐20FEV1, ug	1009 (1109)	1104 (997)	0.675	2068 (830)	**<0.001**
Significant hyper‐responsiveness^b^	29 (58%)	15 (38%)	0.067	6 (7.5%)	**<0.001**
FeNO, (ppb)	29.3 (26.3)	26.7 (24.0)	0.642	‐	
FeNO min 35ppb	16 (32)	10 (26)	0.513	‐	

*Notes*: The data represents the means (standard deviations) or the number (percentages) of children. Calculated using chi‐square test, Fisher's exact test, independent samples *t*‐test and Kruskal‐Wallis test, as appropriate. Bold *p*‐values indicate *p* < 0.05.

Abbreviations: FeNO, exhaled lower respiratory nitric oxide; FEV1, forced expiratory volume in 1 second; FVC, forced vital capacity; PD‐20FEV1, The cumulative dose of methacholine (ug) causing a 20% fall in FEV1.

^a^ Obstruction in spirometry defined as 88% below the reference value of the FEV1/FVC ratio.

^b^ Significant hyperresponsiveness defined as PD‐20FEV1 of the maximum 600 μg of methacholine.

### 3.5 Asthma control during oral immunotherapy

Based on the examination of medical records, OIT did not affect asthma control in the 40 children with regular asthma medication. During the 12 months before starting OIT, one child had one asthma‐related emergency room visit, and during the 12 months after starting OIT there was one emergency room visit in another child. None of the children needed to be admitted for inpatient treatment 12 months before or after starting OIT. The children with asthma showed no differences in lung function, measured in FEV1 [94.9% (12.1) versus 93.1% (10.1), *p* = 0.160], FeNO [22.7 ppb (18.2) versus 22.5 (16.3), *p* = 0.962), or BHR before or after 6–12 months of OIT [PD20FEV1 855 μg (950) versus 975 μg (1050), *p* = 0.390.

## 4 DISCUSSION

We compared BHR and lung function in 89 school‐aged children with persistent egg or peanut allergy and 80 non‐food‐allergic controls and assessed whether OIT for egg or peanut allergy affects asthma control. Three key findings emerged from this study. First, BHR was significantly more common in children with persistent food allergy than in non‐food‐allergic controls regardless of allergy to egg or peanut or the presence of asthma. Second, lung function or BHR did not associate with the OIT outcome. Third, OIT for egg or peanut allergy had no adverse effect on asthma control in children with regular asthma controller treatment.

We observed significant BHR in 58% of the egg‐allergic and 38% of the peanut‐allergic children. Of them, 47% had asthma and 53% were asymptomatic These results are consistent with earlier studies describing increased BHR in children with food allergy compared to healthy controls.^9^, ^10^, ^11^ A study from Poland^10^ demonstrated asymptomatic BHR in 47% of 32 non‐asthmatic children with food allergy. In contrast to our study, they detected BHR in all 22 children who had both food allergy and asthma. In a Finnish study,^9^ 86 children with IgE‐mediated cow's milk allergy in infancy had higher levels of FeNO and increased bronchial responsiveness to histamine at school age than healthy controls. In a mouse model of food and respiratory allergy^31^, mice sensitized to both egg white ovalbumin and house dust mite had significantly greater BHR than mice sensitized to one allergen only, suggesting that food allergy primes the immune system to increase its response to inhalant allergens. An international multicenter study^7^ including 467 adults, examined sensitization to 103 allergens with immune solid phase allergen chip (ImmunoCap ISAC). An increasing number of sensitizations associated with asthma, BHR and higher levels of FeNO, but sensitization to foods associated only with increased FeNO. In our study, more than 90% of the children with persistent food allergy were sensitized to at least two aeroallergens in SPT and 73% had allergic rhinitis. However, this did not correlate with BHR in methacholine challenge testing.

In our patients, DBPCFC elicited respiratory symptoms in 18% of the egg‐allergic and 36% of peanut‐allergic patients. Nevertheless, BHR was less frequent in the peanut‐allergic children, although the difference was statistically insignificant. The prevalence of asthma, baseline lung function, and levels of FeNO were similar in both groups. To our knowledge there are no previous studies comparing peanut and egg allergy in terms of BHR.

Asthma was diagnosed in 50% of the peanut‐allergic and 42% of the egg‐allergic children. This is in line with previous studies where the prevalence of asthma in children with food allergy is approximately 45%–50%.^14^, ^17^ We found significant BHR in less than half of our patients with asthma and the frequency of BHR was no different from the non‐asthmatic children. We showed that OIT to egg or peanut was safe for children with asthma. Their asthma symptom control remained stable and there was no need to step up medication. The number of exacerbations requiring emergency room visits did not increase during OIT. There was no difference in baseline lung function, BHR or FeNO levels before and after OIT, which corresponded well with our previous study, where BHR was measured in children receiving peanut OIT.^23^

Oral immunotherapy was successful in altogether 72 (81%) of our patients after 18 months. We have previously shown that high egg white IgE levels and polysensitization to Gal d 1–4 in egg OIT relate with a slower response and discontinuation.^25^ Here we examined whether sensitization to aeroallergens, baseline lung function, FeNo levels, and the presence of BHR associate with the outcome of OIT to egg or peanut, but we found no correlation.

The strength of this study was a well‐characterized cohort of children with persistent egg or peanut allergy confirmed by DBPCFC. After starting OIT, the patients were followed for 18 months, which provided real‐life confirmation of successful desensitization. We performed post‐OIT methacholine challenges only in 34 patients with asthma, and due to limited resources, the time varied from 6 to 12 months. We did not, however, observe any significant differences in BHR between these timepoints. Additionally, we had access only to asthma follow‐up and emergency room visits, but not to possible family doctor visits. Although we assessed asthma symptom control during each visit, it is possible that some asthma exacerbations remained unnoticed.

In conclusion, our study shows that BHR occurs more frequently in children with persistent egg and peanut allergy than in non‐food‐allergic controls. Lung function and BHR remain stable during OIT and do not predict the outcome. We do not recommend assessing BHR before starting OIT in children without asthma. The finding that OIT is safe for children with asthma is of particular interest as this area has been under‐researched in the past.

## AUTHOR CONTRIBUTIONS

Janne Burman and Mika J. Mäkelä have written the study protocol. Data were collected by Kati Palosuo, Kaarina Kukkonen, and Sami Remes. Janne Burman has made the analysis. Pekka Malmberg has made the pulmonary function test protocols. Anna Pelkonen and Mika J. Mäkelä have supervised the study. Janne Burman and Kati Palosuo have drafted and critically reviewed the manuscript and approved the version to be published and agree to be accountable for all aspects of the work related to its accuracy and integrity.

## CONFLICTS OF INTEREST

The authors declare that there is no conflict of interest that could be perceived as prejudicing the impartiality of the research reported.

## IMPACT STATEMENT

Children with persistent egg or peanut allergy had BHR in methacholine challenge test more frequently than children without food allergy. Oral immunotherapy for egg or peanut allergy was safe for food‐allergic children with asthma requiring regular controller treatment. Oral immunotherapy did not affect their lung function, the degree of BHR, the level of exhaled nitric oxide, or the number of acute asthma‐related emergency hospital visits. Based on our study, we do not recommend assessing BHR before starting OIT in children without asthma.

## Data Availability

The individual participant data collected during the trial are not available for sharing.
